# Effectiveness and cost effectiveness of a stress management training for leaders of small and medium sized enterprises – study protocol for a randomized controlled-trial

**DOI:** 10.1186/s12889-021-10398-4

**Published:** 2021-03-08

**Authors:** J. A. M. Lehmann, E. Schwarz, Z. Rahmani Azad, S. Gritzka, T. Seifried-Dübon, M. Diebig, M. Gast, R. Kilian, U. Nater, M. Jarczok, F. Kessemeier, S. Braun, E. Balint, E. Rothermund, F. Junne, P. Angerer, H. Gündel

**Affiliations:** 1 Department of Psychosomatic Medicine and Psychotherapy, Ulm University Medical Center, Albert-Einstein-Allee 23, 89081 Ulm, Germany; 2 Department of Psychosomatic Medicine and Psychotherapy, University Hospital Tübingen Osianderstraße 5, 72076 Tübingen, Germany; 3 Institute of Occupational and Social Medicine, Heinrich-Heine-University Düsseldorf, Universitätsstraße 1, 40225 Düsseldorf, Germany; 4 Department of Psychiatry II, Ulm University and Bezirkskrankenhaus Günzburg, Ludwig-Heilmeyer-Str. 2, 89312 Günzburg, Germany; 5 Department for Clinical and Health Psychology, Wien University, Liebiggasse 5, 1010 Wien, Austria; 6 Section of Health Care Research and Rehabilitation Research, Faculty of Medicine, University of Freiburg, Breisacher Str. 153, 79110 Freiburg, Germany

**Keywords:** Stress, Stress management, Mental health, Stress management training, Leaders, Well-being

## Abstract

**Background:**

Leaders in small and medium-sized enterprises (SMEs) are exposed to increased stress as a result of a range of challenges. Moreover, they rarely have the opportunity to participate in stress management trainings. Therefore, KMU-GO (ger: Kleine und mittlere Unternehmen – Gesundheitsoffensive; en: small and medium-sized enterprises – health campaign) aims at conducting and evaluating such a stress management training. The focus of evaluation does not only lie on the effects on leaders participating but also on their employees.

**Methods:**

The study is planned as a 2 × 3 mixed design with two groups (intervention and waiting control group) as a between factor and point in time (at baseline, 6 and 12 months later) as a within factor. We aim at collecting data from *N* = 200 leaders. Based on the results of a preceding assessment, an already successfully implemented stress management training was adapted to SME needs and now serves as the framework of this intervention. The stress management training comprises one and a half days and is followed by two booster sessions (each 180 min) about 3 and 6 months after the training. The main focus of this intervention lies on specifying leaders stress reactivity while at the same time investigating its effects on employees’ mental health. Further dependent variables are leaders´ depression and anxiety scores, effort-reward imbalance, sick days and psychophysiological measures of heart rate variability, hair cortisol, and salivary alpha-amylase. Cost-effectiveness analyses will be conducted from a societal and employers’ point of view.

**Discussion:**

Stress management is a highly relevant issue for leaders in SMEs. By providing an adequate occupational stress management training, we expect to improve leaders´ and also employees` mental health, thereby preventing economic losses for SMEs and the national economy. However, collecting data from employees about the success of a stress management training of their leader is a highly sensitive topic. It requires a carefully planned proceeding ensuring for example a high degree of transparency, anonymity, and providing team incentives.

**Trial registration:**

The KMU-GO trial is registered at the German Clinical Trial Register (DRKS): DRKS00023457 (05.11.2020)

## Background

Holding a leadership position comes along with increased responsibility and high job demands. Leaders need to make decisions for the benefit of their company and, at the same time, to ensure that performance and satisfaction of their employees remain high. Employees in leadership positions are thus easily exposed to high levels of occupational stress [1] putting them at risk for the adverse health effects of prolonged stress exposure (cf. [2]). On the organizational level, high levels of work stress may impair job performance [3, 4] as well as job satisfaction [5] and may increase absenteeism and turnover intention [6, 7]. Due to its negative effects on staff turnover and productivity occupational stress is estimated to cause economic losses for companies and the national economy amounting to EUR 617 billion € per year in the EU [8]. For the individual leader, the risk to develop stress-associated diseases such as cardiovascular diseases [9], coronary heart diseases [10], pain [11], depression [12] and burn-out [13, 14] is elevated. Potential mechanisms include stress induced dysregulation of autonomic [15, 16], hormonal [17] and metabolic systems (Jarczok et al. 2013, [18]). But also personality and health behaviour play a role [19].

Besides being stress-impaired themselves, high work stress among leaders may also impact the mental health, job satisfaction and well-being of their employees in short and long-term. For example, Schmidt et al. [20] could show in a 10-year follow-up that a lack of supportive leadership behaviour predicted suboptimal self-rated health independent of job strain n a large general population sample. Similarly, Franke et al. [21] suggest that leaders influence their employees’ health in multiple ways, by having a role model function, through their own stress, their leadership style, and by shaping working conditions. First, highly stressed leaders are more likely to exert pressure onto their staff and elicit distress and strain in their employees, a phenomenon known as ‘emotional contagion’ [22–24]. Second, high stress levels may impede leaders from exerting a stress-preventive leadership style [25]. Instead, they might adopt a rather negative or destructive leading style [26]. Third, implementing stress-preventive working conditions may be less of a priority when leaders experience high stress themselves.

This is especially relevant in small and medium-sized enterprises (SMEs) since leaders may be exposed to particularly high stress and work load: Managerial decisions in SMEs are often made by one person only or a small number of people on the management board [27]. The effects of such decisions are often more immediate and high stakes than in large enterprises, which may offset financial losses caused by unfavourable decisions against other revenue sources [28]. In SMEs, personnel is limited and thus positions often come with multiple roles and broad task responsibilities as opposed to a narrower and more specialized division of work in large companies [29]. Leaders in SMEs have no or only few co-workers on the same hierarchy level and therefore have less opportunity to consult and exchange views with their peers for support (Thorpe et al., 2009). In addition, the personal identification with the job is higher in SMEs, especially in family-led corporations or own enterprises, and boundaries between personal and professional lives tend to blur [30]. This might exacerbate the risk that work stress continues to dwell during after-work hours and may impede recovery and relaxation during the free time [31].

Skillful leadership and stress management competencies can help prevent excessive occupational stress which not only is important for the companies proximal goals (employees satisfaction and absenteeism), but also mitigates the risk for stress-related health issues [23]. Improved stress coping skills in leaders have multiplier effects as they improve leaders’ stress reactions and reduces their employees exposure to work stress [32]. This creates the need for effective, empirically evaluated stress management trainings [33].

Ideally, stress-prevention measures target both, individual and organization-related approaches [34, 35]. Integrated approaches tackle work-related stress on a behavioural level equipping employees to best react and cope with stressors. Simultaneously, they aim to improve organizational structures, and foster health-promoting working conditions and company cultures. In the short-term, however, organizational level prevention measures are more difficult to practically implement than individual-level approaches [36]. In the empirical literature, most stress management interventions thus address behaviour change that can be employed on the individual level. Interventions that use methods derived from cognitive behavioural therapy show the best empirical support or effectiveness on reduction of perceived stress and depressive symptoms [34, 37].

When implementing such stress intervention programs, large companies seem to be better equipped than SMEs when it comes to the required resources and structures [38]. However, in SMEs, the supply of such interventions and tools is much more limited [28]. With the majority of all German companies belonging to the SME-sector (99.3%) and most of the workforce working in SMEs (61.2% [39]), it is important to close this gap and to provide low-threshold trainings that target effective leadership and stress-management [40]. However, research on stress prevention and well-being interventions that target SMEs specifically are scarce [41]. In their meta-analysis, Gerhardt and colleagues call for more research on stress prevention in SMEs and conclude from existing trials that individual-related approaches showed the most promising results to reduce stress at work (2019). In sum, leaders in SMEs are at a particular risk of excessive work stress and its adverse consequences but have been neglected in stress intervention research [42, 43]. We expect that this target group might particularly benefit from stress management trainings and that the demand for such prevention programs might be high.

Beyond its direct impact on the health of leaders and co-workers, worksite mental health interventions are also expected to prevent economic losses to the individual company and the national economy at a whole. At the company level economic effects can be achieved through the reduction of absenteeism and staff turnover, while at the societal level the prevention of chronic disability, long-term unemployment and early retirement are particularly important [8, 44–47].

One intervention that showed prolonged beneficial effects on leaders’ perceived stress is the MAN-GO stress-management training that was conducted in a large steel manufacturing company comprising only male leaders [48]. The intervention reduced perceived stress among leaders and continued to show beneficial effects even after a nine-year follow-up evaluation [49]. The purpose of the present study is also to replicate the findings of the previous longitudinal MAN-GO intervention study and to prove whether its effectiveness generalizes to the SME sector, all genders and all industries. To this end, we adapted the original manual from the MAN-GO training in order to target leaders of both sexes in SMEs. The one and a half days stress management training includes elements from cognitive behavioural therapy, psychodynamic psychotherapy, psychoeducation and mindfulness components and is followed by two booster session (each 180 min) three and 6 months afterwards.

### Aims and objectives

In analogy to the original research design, we will assess self-reported perceived stress and reactivity to stress and symptoms of anxiety and depression as the main outcome variables. To complement the subjective measures, physiological markers of stress (resting blood pressure, indicators of heart rate variability, hair cortisol levels and salivary alpha-amylase) will be measured in the leaders. Assuming that leadership behaviour and leaders’ individual stress levels impact the work stress of their employees, we will assess the effects of the intervention on the leaders’ employees as well. Therefore, employees will rate their own perceived work stress and the leadership behaviour of their superior on questionnaires before and after the stress intervention. Furthermore, the intervention will be evaluated in the light of its economic and organizational benefits: secondary evaluation criteria will therefore be number of sick days, physiological health, and absenteeism, the relationship between leaders and employees as assessed by leaders and employees, and their perception of working conditions.

The study is designed as a randomized controlled trial with a waiting control group evaluating the effectiveness of a stress management training at 6 and 12-months-follow-up measurement time points.

In short, the main objectives of KMU-GO are:

1. To investigate the effectiveness of a stress management training for leaders based on their subjectively perceived mental health and objective physiological markers of stress as well as other outcome variables such as anxiety and depressive or psychosomatic symptoms or sick days in comparison with a waiting control group.
2. To investigate indirect effects on the subjectively perceived mental health of the employees of the trained leaders as well as on their emotional and cognitive irritation, well-being, perception of working conditions and the quality of leader-employee relationship.
3. To evaluate the cost-utility of the stress management training from a societal perspective.
4. To replicate the effects of the previous MAN-GO [48] study, thereby expanding the results to all genders, the SME sector and all industries.
5. To additionally evaluate the cost-effectiveness of the stress management training at the company and societal level

### Trial design

**Fig. 1:**
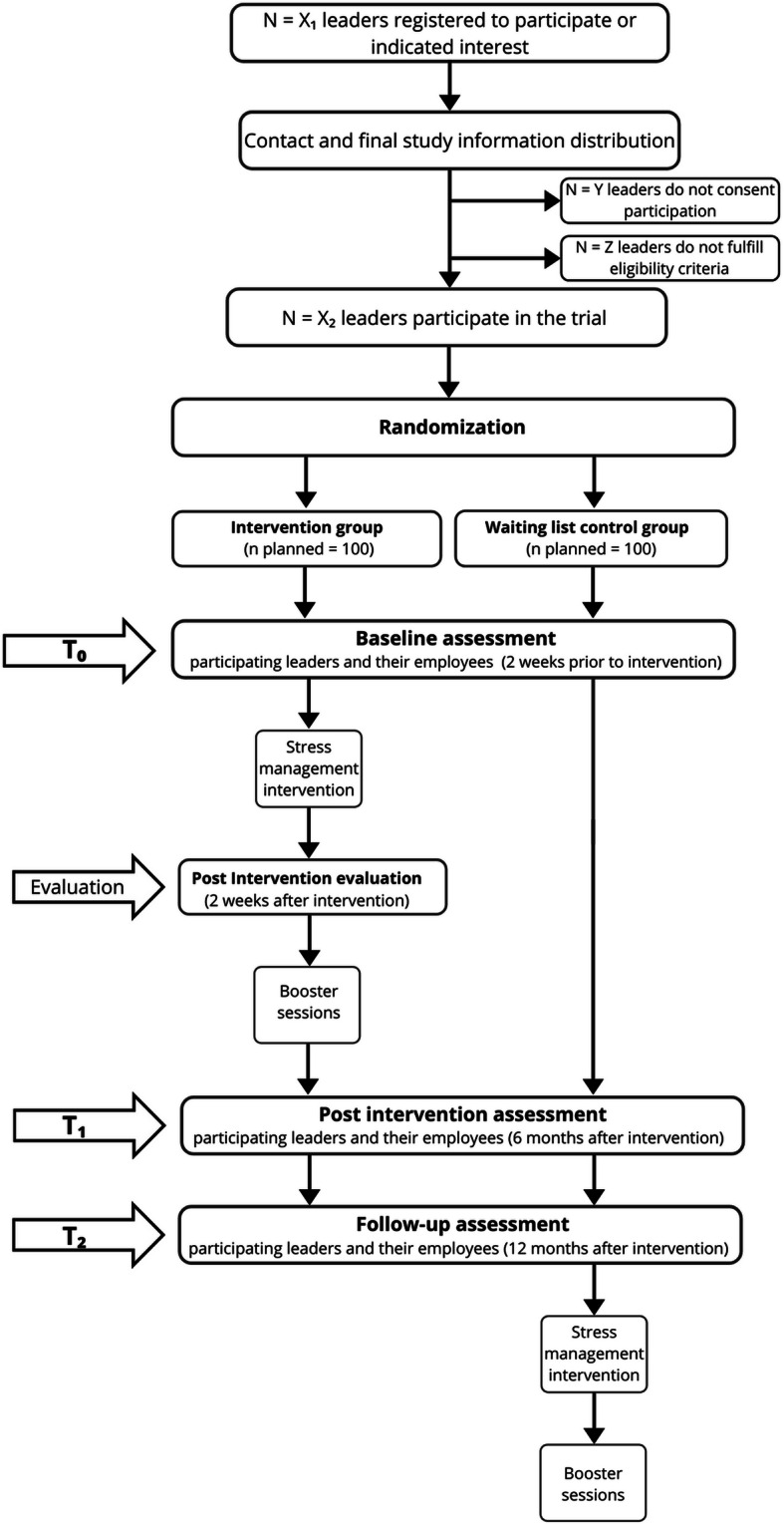
Trial Design

The study is designed as a randomized 2 × 3-mixed design (factor 1 (group): intervention or waiting control group; factor 2 (point in time): baseline, 6 months and 12 months later; see Fig. 1). All participating leaders are going to be randomly assigned to one group. Baseline measures of participants of the intervention group are going to be collected about 2 weeks before their group intervention takes place. Baseline measures of participants of the waiting control group are going to be collected simultaneously. The intervention group is going to take part in the stress management training, followed by two booster sessions after 3 and 6 months. Participant’s satisfaction with the intervention is planned to be assessed within 2 weeks after the intervention. After 6 and 12 months, all relevant outcome variables are planned to be measured (for more detailed information about the outcome variables, please see Section 2.3). The waiting control group will also be measured for all relevant outcome variables after 6 and 12 months, but is going to take part in the intervention only after the last measurement. Employees of the participating leaders will be measured at the same points in time: baseline, 6 and 12 months later.

## Method

### Population and sample size

#### Leaders

The target group are leaders of small and medium-sized enterprises (SMEs). We define SMEs as enterprises with up to 500 employees [50, 51] without regarding the annual sales. Leaders of all genders aged between 18 and 65 years are allowed to participate as long as they are not about to retire within the next year. A sample size of *n* = 100 participants in the intervention group and *n* = 100 participants in the waiting control group is planned (*N* = 200 participants).

The sample size calculation was performed using G*Power3.1. Based on a 2 × 3-mixed design (factor 1 (group): intervention or waiting control group; factor 2 (point in time): baseline, 6 months and 12 months later) the required sample size for a MANOVA with measurement repetition was estimated using the following predefined parameters: mean effect size f = .25 (Ω = 0.06), α-error = .05, test strength 1-β = .80. This results in a required sample size of *n* = 158 participants (*n* = 59 per group). The calculation of the required sample size was based on the procedure proposed by Prajapati et al. (2010) to calculate the required sample sizes for all possible effects (within, between, interaction) of the present design and to use the largest calculated sample size. Due to the high effort of the outcome criteria to be collected (e.g., saliva samples, 24-h heart rate variability) a high lost-to-follow-up as well as numerous missing values can be expected. We therefore calculate with a total failure rate of 21% (lost-to-follow-up and due to missing values), resulting in a required sample size of *N* = 200 (*n* = 100 per group at T_0_).

Recruitment is carried out in cooperation with AOK Baden-Württemberg, IHK Ulm, IHK Reutlingen, Offensive Mittelstand, Handwerkskammer Ulm, and Südwestmetall. Flyers and advertising texts will be sent via email through suitable channels and presented at suitable events (face-to-face and online). Leaders who want to participate in this study have to be aged between 18 and 64 years, need to sign the informed consent (see appendix), and must have sufficient German language skills. Due to the variety and size of the above mentioned cooperation partners, the achievement of the targeted sample size can be considered as realistic. Moreover, leaders will be reminded by email to take part in the follow-up measures. As the participation is voluntary, consent may be withdrawn without any reason. The protocol was approved by the institutional ethics committee.

#### Employees

In addition to the leaders participating in the intervention, we also aim to at surveying their employees in order to gather data on the indirect effects of the intervention. Thus, leaders will be informed in advance about the additionally planned and voluntary survey of their employees. Upon prior written consent, leaders will receive information material in order to inform their employees adequately about the study and their ways to participate: (1) qualitative serial interview at T0 and T2 (only intervention group) and/or (2) online survey at T0, T1 and T2. Due to the so far unknown team size, a sample size calculation pertaining to the online survey cannot be performed. On average, we expect three employees per leader to fill in the questionnaires, resulting in a total of *N* = 300 employees per group. Regarding the qualitative serial interviews, we aim to interview *N* = 25 employees (intervention group only) at T0. Thus, we consider a conservative drop-out rate of 40% with the aim of being able to interview *N* = 15 employees at T2.

The randomization of employees is based on the group allocation of their leaders (convenience sampling). Employees of leaders who want to participate in this study have to be aged between 18 and 65 years, need to sign the informed consent, and must have sufficient German language skills in order to complete the online questionnaire or to be interviewed, respectively.

Although leaders can decide on their own whether to involve their employees or not, we will encourage leaders to do so by highlighting the importance of employees’ inclusion. Moreover, we offer anonymous employee summary reports after T2 to each leader, if the following conditions are met: (1) Their employees agree to the anonymous disclosure of results in a summary form (last question in online survey) and (2) ≥ 5 employees per leader provide data.

First, employees will be informed by their leader, preferably in 1-on-1 or small group meetings. Here, employees will also receive written information about the conditions of participation. Second, direct contact is established between employees and study team in order to ask further questions or gain additional information, if necessary. Following verbal and written information, employees can decide voluntarily whether to participate. All participants at T0 are going to be reminded by email to participate again at the follow-up measures. As the participation is voluntary, consent may be withdrawn without reasons that need to be disclosed.

### Intervention

The implemented stress management training is based on the successfully conducted intervention of the MAN-GO project [48]. It is going to be adapted based on the results of a previous (and still ongoing) needs analysis concerning format and content. Depending on the results, it will most likely obtain the following modules:

- Stress: effects of stress, individual stress symptoms, mentalization ability under stress
- Work stress models: gratification model, demand-control-model, organizational fairness
- Leadership: stress-preventive, health-oriented, and relational leadership, leaders as role models, new demands on leaders based on COVID-19 and the digitalization
- Short term relief: brooding strategies, attention guiding
- Conflict management: dealing with (emotional) conflicts and catch-22 situations at the workplace
- Relaxation: progressive muscle relaxation, 1-min meditation
- Resources: sweet spot imagination, social support, 10 fingers
- Case work: working on cases brought in by participants, approach based on Balint groups and collegial case advise
- Optional additive online modules: impact of stress on cardiovascular diseases, further stress symptoms

Trainings are going to take place in groups with 8–12 leaders and are held by two trainers. As far as possible in the context of COVID-19, they should be carried out in persona. Concerning the didactical frame, the trainings consist of a mixture of lectures, open discussions as well as single, peer, and group work. Trainings will take place in Ulm and surrounding as well as in Tuebingen and surrounding.

### Outcome variables

All measurements will take place in Germany. More specifically, participants are recruited in Ulm and Tuebingen and their surroundings. Participants are allowed to answer all questions from any suitable place, as questionnaires and further items are provided online. For the physiological measures (leaders only), appointments in Ulm and Tuebingen are going to be arranged.

#### Leaders

Leaders are going to be asked to complete single items, 9 questionnaires and three physiological measures.

##### Single items

*Sociodemographic data.* Participants are going to be asked for their gender, age, marital status, children living in the household and the highest educational level attained. Furthermore, the monthly net income can be entered voluntarily and the number of personal dependants.

*Work-related data.* Regarding the professional situation, it is asked to indicate the branch, current position, need to do shift work and number of employees to be supervised. Furthermore, the average working time and break times as well as unpaid overtime are enquired.

*Health-related data.* Days of incapacity to work in the last 3 or 12 months, activity in everyday working life, weight and health behaviour (cigarette and alcohol consumption) are going to be recorded.

##### Questionnaires

All subsequent questionnaires are collected at three measurement times (T_0_, T_1_, T_2_). The target outcome is the change in the intervention group compared to the waiting control group over time.

*Perceived Stress-Reactivity-Scale* [52]. The PSRS assesses perceived stress reactivity on 5 scales: reactivity to social assessment (5 items, “If I am wrongly criticized by others ...”), reactivity to failure (4 items, e.g., “If I did something wrong ...”), reactivity to social conflict (5 items, e.g., “When I argued with other people ...“), reactivity to work overload (5 items, e.g., “When I have little time for my work ...”) and prolonged reactivity (4 items, e.g., “When I have free time after strenuous work ...“). In addition, an overall score can also be calculated. All items are answered by agreeing with one of three default answers. Scale reliability is sufficiently high and varies between α = .79 and α = .82 with α = .89 for the total score (Cronbach’s Alpha).

*Effort Reward Imbalance Questionnaire (ERI,* [53]). The ERI conducts self-assessments of psychosocial workloads on three scales: exertion (6 items, e.g., “I am often interrupted and disturbed in my work”), reward (11 items, e.g., “I get the recognition I deserve from my superiors”) and over-commitment (6 items, e.g., “Those who are closest to me say I sacrifice myself too much for my job”). Reward is a multidimensional concept and consists of the following subscales: recognition, pay/progression and job security. The exertion and reward dimensions are assessed with five-point Likert-scale, ranging from “unexposed to adverse condition” (1) to “exposed to adverse conditions and very distressed” (5). Over-commitment is measured with a four-point Likert-scale, ranging from “Strongly disagree” (1) to “Strongly agree” (4). The internal consistency of the scales is sufficiently high and between α = .62 and α = .90 (Cronbach’s Alpha).

*Hospital Anxiety and Depression Scale (HADS, Herrmann, 1997).* The HADS is a 14-item questionnaire (7 items each for anxiety and depression) for self-assessment of depressive (e.g., “I still enjoy the things I used to enjoy”) and anxiety symptoms (e.g., “Worrying thoughts go through my mind”). All items are answered by agreeing with one of four default answers. Scale reliability is high with α = .86 for anxiety and α = .91 for depression (Cronbach’s Alpha).

*Leader-Member Exchange 7 (LMX-7, Graen & Uhl-Blen, 1995).* The LMX-7 questionnaire consists of 7 items assessing the relationship between leaders and employees (e.g., “How well do you understand the work-related problems and needs of your employees?”) on a 5-point Likert-Scale. Cronbach’s Alpha is high: α = .84 [54].

*Occupational Self-Efficacy Expectations – short scale (*Berufliche Selbstwirksamkeits-erwartung, BSWE*, Schyns & von Collani, 2014).* The BSWE *–* short scale consists of 8 items measuring occupational self-efficacy expectations (e.g., “When unexpected situations arise at work, I always know how to act”) with a 6-point Likert-Scale ranging from “completely true” (1) to “not true at all” (6). Scale reliability is high with α = .84 (Cronbach’s Alpha).

*Psychosocial Safety Climate Questionnaire 4 (PSC-4,* [55]). The PSC-4 is a short questionnaire with only 4 items measuring the Psychosocial Safety Climate at work (e.g., “Senior management show support for stress prevention through involvement and commitment”). All items are answered using a 5-point Likert-Scale, ranging from “strongly disagree” (1) to “strongly agree” (5).

*Operationalized psychodynamic diagnosis structure questionnaire (OPD-SF, Ehrental* et al.*, 2012).* The OPD-SF assesses the Axis 4 (Structure) from the Operationalized Psychodynamic Diagnostic system (OPD). Only two of the five dimensions are used for the purpose of this study: self-awareness (12 items, e.g., “I often have feelings that I cannot understand”) and self-regulation (13 items, e.g., “When I’m very upset, I often act rashly”). Items are measured on a five-point Likert-scale ranging from “strongly disagree” (1) to “strongly agree” (5). Cronbachs α ranges from .88 to 91 (self-awareness) and .82 to .84 (self-regulation) [56].

*Client Sociodemographic and Service Receipt Inventory (CSSRI,* [57]). The use and costs of health care resources will be estimated by means of the CSSRI adapted for the application in the target population [57].

*EuroQol’s questionnaire for health related quality of life (EQ-5D 5 L,*[58]*).* Health-related quality of life will be assessed by means of the EuroQol EQ-5D 5 L. The EQ-5D 5 L defines subjective health states at the following five dimensions Mobility, Self-care, Usual activities, Pain and discomfort, Anxiety and depression at a five-level scale [58]. The individual health states will be evaluated by means of country-specific representative preference based value sets [59].

##### Physiological measures

*Hair cortisol.* As a first biological stress parameter, the content of hair cortisol (determined via a 2 cm long hair strand) as an indicator for the hypothalamic-pituitary-adrenal axis is going to be collected. Several thin hair strands will be cut as close as possible to the scalp from the posterior vertex region of the head. For determination of hair cortisol concentrations, the first scalp-near 2 cm segment will be used which is thought to reflect the cumulative cortisol secretion of the past 2 months [60]. Hair wash and cortisol extraction procedures are based on a laboratory protocol by Stalder et al. [61], with minor modifications. In brief, hair samples will be washed twice for 3 min using 3 mL isopropanol. For cortisol extraction, 10 ± .5 mg whole, finely cut hair will be incubated in 1.8 mL methanol for 18 h at room temperature. After incubation 1.6 mL will be transferred in another glass vial. Then, 1.6 mL of the supernatant is evaporated at 50 °C until samples are completely dried. Finally, the samples will be resuspended with 225 μL HPLC ultra-pure water and vortexed for 20 s. For cortisol determination, a commercially available cortisol luminescence immunoassay will be used (LIA; IBL International, a Tecan Group company, Hamburg, Germany).

*Saliva alpha-amylase.* As a second biological stress parameter, alpha-amylase activity in saliva is measured as basal activity of the sympathetic nervous system [62, 63]. Using SaliCaps (IBL International, a Tecan Group company, Hamburg, Germany), participants will be asked to not swallow for 2 min and then transfer all accumulated saliva in pre-labelled vials. Furthermore, they will be asked to provide information on meals and drinks 2 h prior to saliva collection in order to control for confounding variables. The saliva samples will be stored as cool as possible in the participants’ fridge or freezer and are then at the earliest convenience stored at − 80 °C in freezers of the University. Salvia alpha-amylase activity will be measured using a kinetic colori metric test (for details, see also [64]) and reagents obtained from DiaSys Diagnostic Systems (Holzheim, Germany). Saliva will be diluted 1:400 using 0.9% saline solution. The reagents contain the enzyme alpha-amylase in a specified amount and alpha glucosidase, which converts the substrate ethylidene nitrophenyl to p-nitrophenol. The rate of formation of p-nitrophenol is directly proportional to the samples’ amylase activity and is detected using an absorbance reader at 405 nm (Biotek Synergy HTX, BioTek Instruments, Winooski, VA, USA).

*Heart rate variability.* A 24-h measurement of heart rate variability is collected using a Faros 180 ECG monitor with a textile belt. Circadian parameters of 24 HRV measures will serve as an indicator of mental health and stress perception ([65, 66]; Williams et al., 2018).

*Satisfaction with the training.* Participants are going to be asked to evaluate their trainers (8 questions each, 6-point Likert-scale) and the training (12 questions, 6-point Likert-scale) concerning acceptance and satisfaction.

#### Employees

Regarding the assessment of employees, seven validated questionnaires and various single-item measures will be used. Employees receive an email with a link that leads to the described questionnaires. Depending on how they wish to proceed or what is most convenient for the employees, they can fill it in at home or at work.

##### Single items

*Socio-demographic data.* The data collected includes information about gender, age, marital status, the highest educational level attained, the number of years worked under the current leader, organizational tenure and hours of direct contact with leader per week.

*Change-related data. P*articipants will be asked to rate the statement “I have noticed a change in my leader’s behavior after the training.” This item will be assessed on a 5-point Likert scale ranging from 1 “do not agree” to 5 “fully agree”; respectively there will be another option “My leader will take part in the training in 2022”. This item will only be used at T1 and T2.”.

At the end of the survey participants will have to answer two additional questions. At first, they have to indicate their seriousness during participation [67]. Therefore, we will directly ask participants if questions were answered in a serious manner (“Please indicate whether you have been actively involved in the survey, so that we can use your answers for our scientific analysis, or whether you were just clicking through to gain a first impression?”). Second, employees can decide, whether their answers can be used for a de-identified employee summary report, which will be provided for the leaders after the completion of T2.

##### Questionnaires

*Short questionnaire for work analysis* (ger.: Kurzfragebogen zur Arbeitsanalyse, KFZA, [68]). Working conditions will be assessed by the KFZA, which consists of eleven scales derived from 26 items. These scales comprise Decision latitude, Variety of work, Task identity, Social support, Teamwork, Workload (qualitative), Workload (quantitative), Interruption of work, Environmental stress, Information and codetermination, Company benefits. Sample items are: “How much influence do you have on what work is assigned to you?” and “I am often under time pressure”. All items are measured on a 5-point Likert scale ranging from 1 “does not apply at all” to 5 “is completely true”. The internal consistency of the scales is set between .40 and .76 (Cronbach’s α); in studies with larger samples Cronbach’s α ranged from .63 to .80 [69].

*Cognitive and emotional irritation scale* (IS [70]). The IS measures perceived cognitive and emotional strain. Irritation refers to a psychological state of impairment due to the experience of goal-discrepancy. The IS consist of 8 items that have to be answered on a 7-point Likert scale resulting in two first-order factors: First, Rumination (e.g., “Even when I am on vacation, I sometimes have to think about problems at work”) invokes increased efforts in order to achieve goals, whereas the second factor Irritability (e.g., “I get angry easily”) reflects a tendency to lose the incentive to pursue a certain goal. Internal consistency scores range from .85 to .97 for both subscales.

*Subjective psychological well-being* (WHO-5, Bech et al., 2003). Employees’ well-being will be measured using the five-item World Health Organization Well-Being Index (WHO-5) representing the most widely used questionnaire for measuring this construct [71]. Items are assessed on a 6-point Likert scale, ranging from 0 “at no time” to 5 “all of the time” concerning the last 2 weeks (e.g., “… I have felt calm and relaxed”). The raw score is multiplied by 4 resulting in a final score ranging from 0 to 100 (representing the best possible well-being). The WHO-5 is suitable for depression screening. The split-half reliability (Guttman’s coefficient) is r_tt_ = .87 (Brähler et al., 2007).

*Effort Reward Imbalance* (ERI, [53]) and *Relationship between employees and their managers* (LMX-7, Schyns & Paul, 2014). Both questionnaires are already described in section 2.3.1.

*Serial qualitative interviews in the intervention group*. We will conduct serial qualitative interviews at T_0_ and T_2_ as interviewing participants on multiple occasions is useful in understanding longitudinal change, thus variation in behaviours or circumstances over time [72]. Particularly, semi-structured interviews with employees will be used in order to understand the delivery and impact of indirect outcomes on employees following their leaders‘participation in the intervention. Thus, employees will be asked to describe the leadership behaviour, the leader’s stress level, leadership styles, working conditions and self-perceived health in a narrative style. Following a qualitative content analysis [73] the preliminary category system developed at T_0_ will be revisited at T_2_ in order to identify potential changes.

### Timeline

The total trial duration is 12 months for the intervention group, and 18 months for the control group. Baseline data of all leaders and employees is going to be collected in the beginning (spring 2021). The intervention group participates in the stress management training in the weeks thereafter (duration: 1.5 days) followed by two booster sessions 3 and 6 months later, each lasting for 180 min. All participants are going to be measured again after 6 and 12 months. Afterwards, participants of the waiting control group are going to participate in the training, followed by the two booster sessions 3 and 6 month thereafter.

### Assignment of interventions

Each single participant will randomly be allocated to the intervention or waiting control group. A study staff member uses computer-generated random numbers for the allocation of the participants. It is not possible to blind participants about their group affiliation, due to the different intervention times.

### Data collection, management, and analysis

*Data collection.* Leaders’ sociodemographic and work-related data will be assessed at baseline before the intervention. The self-report measures of health-related indices, perceived stress (PSRS-23 [52]), work satisfaction (ERI [53]), symptoms of depression and anxiety (HADS-D [74]) and well-being (WHO-5 [75]) will be assessed at three different points of time: at baseline, at a 6-month-follow-up post-intervention and a 1-year-follow-up. The questionnaires can be filled in at home or during the clinic visits, where the physiological data will be recorded across the three time points. All questionnaire data will be assessed online via the encrypted German Unipark servers. Physiological measures, i.e. hair cortisol, saliva samples for alpha-amylase activity and blood pressure will be collected by a trained study nurse on site. To assess the 24-h-heart rate variability, participants will wear a belt for 24 h after their appointment at the clinic. Employees will receive online questionnaires that can be filled in during work in order to rate their individual stress levels and their perceived interaction with their leaders. Data will be transmitted electronically across encrypted German servers provided by the online assessment tool Unipark.

#### Data management

All data will be stored pseudonymously. All identifying information of participants will be removed from the datasets. Data will be stored locally on the clinic networks that underlie a strict security protocol to store sensitive clinical data. If data will need to be transferred electronically, it will be password encrypted. Documents with identifying information, such as signed consent to participation will be stored securely and separately from the study data. Only project staff members will have access to the data collected. The staff members are subject to the obligation of secrecy.

#### Statistical methods

Data will be analysed using the statistic software IBM SPSS Statistics 27. Descriptive analyses will be conducted after the baseline. A multivariate analysis of variance (MANOVA) with all relevant outcome parameters as specified above will be calculated in order to test the effects of the intervention on different dependent variables. Potential covariates will be included in the analysis. Therefore, if relevant covariates occur, a MANCOVA will be performed instead of the MANOVA. A covariate will be identified as such, if a demographic variable will correlate with the outcome variable or if there is a significant difference between the intervention group and the waitlist control group across this variable.

To evaluate the effects of the stress management intervention more specifically, changes in outcome variables will be analysed by two-factorial ANOVA with repeated measures and group (intervention vs. waiting control group) as a between-person effect. The core hypothesis is that perceived stress and physiological stress parameters of leaders will significantly decrease from baseline to follow-up measurements for participants in the intervention group. Thus, we will test the interaction effect of time x group for the different outcome variables. Where necessary, Bonferroni correction will be applied to avoid inflation of the type one error by multiple comparisons.

#### Health economic evaluation

Incremental cost-effectiveness analyses (ICEA) by means of the net-benefit approach [76, 77] will be performed from the perspective of SME companies and from the societal perspective. For the SME perspective the incremental costs of the intervention compared to non-intervention incurred by the companies will be related to the incremental reduction in sick-leave days. For the societal perspective the incremental total costs of illness including the direct costs of health care resources and the indirect costs due to productivity losses will be related to the incremental gain in quality adjusted life years (QALYs) [76, 77].

Point estimates of incremental costs effectiveness ratios (ICER) will be computed for both perspectives. Stochastic uncertainty of the ICER will be estimated by means of non-parametric bootstrapping with 4000 replications [76, 78]. ICER will be interpreted on the basis of the cost-effectiveness plane [76]. The significance of the ICER will be estimated on the basis of the cost-effectiveness acceptability curve [76].

Results of the ICEA from the company perspective will provide the necessary maximum willingness to pay for the prevention of one sick leave day at which the intervention is cost-effective at the company level with a probability of 95%. Results of the ICEA from the societal perspective will provide the necessary maximum willingness to pay for the gain of one additional life year in full health (QALY) at which the intervention is cost-effective at the level of the German national economy with a probability of 95% [76].

### Handling of missing and spurious data and drop outs

We do not expect item-wise missing values as all questionnaires will be assembled digitally with notifications if items are missing. However, in the case of missing items for questionnaires the missing item score will be substituted by the mean item score of the remaining questionnaire items.

As participating leaders can withdraw from the physiological assessments, we expect some missing data for the physiological stress measures. In physiological stress assessments, measurement errors may occur. Especially for heart rate variability, which will be measured over a period of 24 h without surveillance of clinical personnel, measurement errors may occur. Thus, all data will be checked for plausibility and carefully tested against potential measurement errors. Participants with missing or spurious data will remain in the sample and will only be excluded for those analyses that involve the missing data.

As the study has a longitudinal design that extends over 1 year in time, some dropouts will be inevitable. We will, however, endeavour to retain as many participants of the original sample and will try to maximize compliance with the study’s data assessments. We accounted for some dropouts in our power analyses. Participants who drop out after t1 (the first follow-up measurement) will be included in the data analyses up to this point in time. An intention-to-treat analysis will be conducted to evaluate whether the sub-sample of dropouts significantly differs from the adherent sample.

### Monitoring

*Data monitoring*. A data monitoring committee is usually formed to monitor the data for potential adverse effects the intervention has on the participants. Since the intervention has no known risk, no data monitoring committee is formed. Furthermore, no interim analysis is conducted and no stopping guidelines are formulated.

*Harms*. The intervention has a low risk of adverse effects. Therefore, no potential harm is recorded. During the intervention sessions participants are monitored by the trainers. If there are unexpected adverse effects, the present trainer takes care of the concerned participant after the training. With regard to the measurements, no risk or complications are expected from measuring blood pressure and recording HRV by using electrodes. If there is a lot of hair on the chest, it may be necessary to remove the hair. However, no permanent damage is to be expected. All experimental procedures used in the study are harmless to health. The only possible risk of becoming aware of sensitive personal data is minimized by pseudonymization and access restrictions.

*Auditing.* No auditing is planned. An ongoing process evaluation takes place within the respective study sites and in the study network as whole. Corrective action will be taken when necessary.

### Ethics and dissemination

#### Consent or assent

Written informed consent is a prerequisite in order to participate. All employees and leaders interested in participation will receive verbal and written information from the study staff prior to the start of the study.

#### Confidentiality

In order to preserve confidentiality, all quantitative data of leaders and their employees will be pseudonymized by a self-generated identification code. The survey data will be stored on password-protected data storage devices. The written informed consent form will be locked in cabinets, respectively. Thus, the identification of an individual person by linking a participant’s name to an identification code will be prevented. Only authorized study team members will have access to original data.

Qualitative interviews with employees will be audio recorded, with the absence of mentioning personal data during interviews. After finishing the audio recording, socio-demographic data of the interview partners will be collected (gender, age, the highest educational level attained, the number of years worked under the current leader, organizational tenure, hours of direct contact with leader per week and number of employees working in the company). Following the data collection phase, the audio recordings will be transcribed guaranteeing complete anonymity by removing last personally identifiable information. The audio recordings of the individual interviews will be erased immediately after transcription. After expiry of the obligation to preserve records, the transcripts will also be deleted. Transcripts will be stored in separate data storage devices with password protection, therefore anonymized data cannot be linked to signed consent forms, original audio files (e.g., before deletion), or sociodemographic data.

Statutory provisions will guide the collection, evaluation and use of study related quantitative and qualitative data (e.g., participants‘voluntary written informed consent before participation).

#### Access to data

During the process of data collection, the database is only accessible to authorized study members bound to secrecy. After database closure, access rights for biometricians regarding the physiological parameters will be granted.

#### Dissemination policy

Study results will be made available to the scientific community, policy makers, health insurances and the SME sector, pursuing a wide dissemination. If desired, study participants will receive a summary of the general study’s research findings. All study results reported will be anonymous.

## Discussion

As already described in the introduction, leaders of SMEs are, in general, exposed to high levels of stress. The current COVID-19 pandemic raised the work-related stress level even more (Hayes et al., 2020). Therefore, it is highly valuable to support these leaders with an appropriate stress management training. As there exists a wide range of offers, it is also of value to proof the effectiveness of such a training. A worthwhile training should lead not only to improved short-term, but also to improved long-term outcomes. Thus, KMU-GO aims at closing a relevant gap by investigating this question.

One big strength of the introduced study design is its variety in outcomes measures. The impact of our intervention is going to be assessed subjectively with questionnaires, objectively with physiological measures and also through external assessment using questionnaires filled in by the employees.

This inclusion of employees of participating leaders is an innovative extension of the original MAN-GO study. Likewise, the qualitative method chosen offers a new approach since a systematic review calls for the expansion of the primarily used quantitative methodology in organizational stress research [79]. A qualitative explanatory perspective might be able to foster the understanding of the origin and development of leaders‘and employees‘relationship and its link to the experience of stress [79]. Despite the study’s innovative character on the employee focus, the inclusion of employees and their participation might face recruitment and retention challenges [80] as well as general response challenges (e.g., Halbesleben & Whitman, 2013; Nesterkind & Ganster, 2012). In order to capture indirect effects of the intervention, employees need to be surveyed at three measurement time points, without taking part in the intervention themselves. Therefore, we are going to take precautions and actions to keep the number of employees participating as high as possible:

- Conducting employee interviews prior to the start of the study itself, exploring circumstances for employee surveys in SME needed to increase feasibility, motivation and participation.
- Ensuring transparency and access to study information as well as a contact person since the assurance of confidentiality is considered a prerequisite for participation.
- Promoting the active support of participating leaders by providing a manual on how and when to advertise the study in a positive light and thereby enhancing the acceptance in their organizations.
- Keeping participating leaders and employees in the loop by communicating next steps.
- Providing team incentives after the completion of the employee survey at T_2_.

This protocol has been drafted in accordance with the SPIRIT guidelines (see Table 1 in Appendix [81]).

## Data Availability

Data and intervention materials will be available from the corresponding author on reasonable request.

## Appendix

**Table 1:** SPIRIT 2013 Checklist

Section/item	Item	Description	Addressed on Page
**Administrative information**			
Title	1	Descriptive title identifying the study design, population, interventions, and, if applicable, trial acronym	1
Trial registration	2a	Trial identifier and registry name. If not yet registered, name of intended registry	2
	2b	All items from the World Health Organization Trial Registration Data Set	-
Protocol version	3	Date and version identifier	-
Funding	4	Sources and types of financial, material, and other support	14
Roles and responsibilities	5a	Names, affiliations, and roles of protocol contributors	1, 14
	5b	Name and contact information for the trial sponsor	14
	5c	Role of study sponsor and funders, if any, in study design; collection, management, analysis, and interpretation of data; writing of the report; and the decision to submit the report for publication, including whether they will have ultimate authority over any of these activities	14
	5d	Composition, roles, and responsibilities of the coordinating centre, steering committee, endpoint adjudication committee, data management team, and other individuals or groups overseeing the trial, if applicable (see Item 21a for data monitoring committee)	10
**Introduction**			
Background and rationale	6a	Description of research question and justification for undertaking the trial, including summary of relevant studies (published and unpublished) examining benefits and harms for each intervention	2-3
	6b	Explanation for choice of comparators	-
Objectives	7	Specific objectives or hypotheses	3-4
Trial design	8	Description of trial design including type of trial (e.g., parallel group, crossover, factorial, single group), allocation ratio, and framework (e.g., superiority, equivalence, noninferiority, exploratory)	4-5
**Methods: Participants, interventions, and outcomes**			
Study setting	9	Description of study settings (e.g., community clinic, academic hospital) and list of countries where data will be collected. Reference to where list of study sites can be obtained	6
Eligibility criteria	10	Inclusion and exclusion criteria for participants. If applicable, eligibility criteria for study centres and individuals who will perform the interventions (e.g., surgeons, psychotherapists)	4
Interventions	11a	Interventions for each group with sufficient detail to allow replication, including how and when they will be administered	6, 9
	11b	Criteria for discontinuing or modifying allocated interventions for a given trial participant (e.g., drug dose change in response to harms, participant request, or improving/worsening disease)	10
	11c	Strategies to improve adherence to intervention protocols, and any procedures for monitoring adherence (e.g., drug tablet return, laboratory tests)	-
	11d	Relevant concomitant care and interventions that are permitted or prohibited during the trial	-
Outcomes	12	Primary, secondary, and other outcomes, including the specific measurement variable (e.g., systolic blood pressure), analysis metric (e.g., change from baseline, final value, time to event), method of aggregation (e.g., median, proportion), and time point for each outcome. Explanation of the clinical relevance of chosen efficacy and harm outcomes is strongly recommended	6-9
Participant timeline	13	Time schedule of enrolment, interventions (including any run-ins and washouts), assessments, and visits for participants. A schematic diagram is highly recommended (see Figure 1)	5
Sample size	14	Estimated number of participants needed to achieve study objectives and how it was determined, including clinical and statistical assumptions supporting any sample size calculations	4
Recruitment	15	Strategies for achieving adequate participant enrolment to reach target sample size	4-6
**Methods: Assignment of interventions (for controlled trials)**			
Allocation:			
Sequence generation	16a	Method of generating the allocation sequence (e.g., computer-generated random numbers), and list of any factors for stratification. To reduce predictability of a random sequence, details of any planned restriction (e.g., blocking) should be provided in a separate document that is unavailable to those who enrol participants or assign interventions	9
Allocation concealment mechanism	16b	Mechanism of implementing the allocation sequence (e.g., central telephone; sequentially numbered, opaque, sealed envelopes), describing any steps to conceal the sequence until interventions are assigned	-
Implementation	16c	Who will generate the allocation sequence, who will enrol participants, and who will assign participants to interventions	9
Blinding (masking)	17a	Who will be blinded after assignment to interventions (e.g., trial participants, care providers, outcome assessors, data analysts), and how	9
	17b	If blinded, circumstances under which unblinding is permissible, and procedure for revealing a participant’s allocated intervention during the trial	-
**Methods: Data collection, management, and analysis**			
Data collection methods	18a	Plans for assessment and collection of outcome, baseline, and other trial data, including any related processes to promote data quality (e.g., duplicate measurements, training of assessors) and a description of study instruments (e.g., questionnaires, laboratory tests) along with their reliability and validity, if known. Reference to where data collection forms can be found, if not in the protocol	9
	18b	Plans to promote participant retention and complete follow-up, including list of any outcome data to be collected for participants who discontinue or deviate from intervention protocols	4-6
Data management	19	Plans for data entry, coding, security, and storage, including any related processes to promote data quality (e.g., double data entry; range checks for data values). Reference to where details of data management procedures can be found, if not in the protocol	9
Statistical methods	20a	Statistical methods for analysing primary and secondary outcomes. Reference to where other details of the statistical analysis plan can be found, if not in the protocol	9-10
	20b	Methods for any additional analyses (e.g., subgroup and adjusted analyses)	
	20c	Definition of analysis population relating to protocol non-adherence (e.g., as randomised analysis), and any statistical methods to handle missing data (e.g., multiple imputation)	
**Methods: Monitoring**			
Data monitoring	21a	Composition of data monitoring committee (DMC); summary of its role and reporting structure; statement of whether it is independent from the sponsor and competing interests; and reference to where further details about its charter can be found, if not in the protocol. Alternatively, an explanation of why a DMC is not needed	10
	21b	Description of any interim analyses and stopping guidelines, including who will have access to these interim results and make the final decision to terminate the trial	10
Harms	22	Plans for collecting, assessing, reporting, and managing solicited and spontaneously reported adverse events and other unintended effects of trial interventions or trial conduct	10
Auditing	23	Frequency and procedures for auditing trial conduct, if any, and whether the process will be independent from investigators and the sponsor	10
**Ethics and dissemination**			
Research ethics approval	24	Plans for seeking research ethics committee/institutional review board (REC/IRB) approval	14
Protocol amendments	25	Plans for communicating important protocol modifications (e.g., changes to eligibility criteria, outcomes, analyses) to relevant parties (e.g., investigators, REC/IRBs, trial participants, trial registries, journals, regulators)	14
Consent or assent	26a	Who will obtain informed consent or assent from potential trial participants or authorised surrogates, and how (see Item 32)	10
	26b	Additional consent provisions for collection and use of participant data and biological specimens in ancillary studies, if applicable	-
Confidentiality	27	How personal information about potential and enrolled participants will be collected, shared, and maintained in order to protect confidentiality before, during, and after the trial	9-11
Declaration of interests	28	Financial and other competing interests for principal investigators for the overall trial and each study site	14
Access to data	29	Statement of who will have access to the final trial dataset, and disclosure of contractual agreements that limit such access for investigators	9
Ancillary and post-trial care	30	Provisions, if any, for ancillary and post-trial care, and for compensation to those who suffer harm from trial participation	-
Dissemination policy	31a	Plans for investigators and sponsor to communicate trial results to participants, healthcare professionals, the public, and other relevant groups (eg, via publication, reporting in results databases, or other data sharing arrangements), including any publication restrictions	11
	31b	Authorship eligibility guidelines and any intended use of professional writers	14
	31c	Plans, if any, for granting public access to the full protocol, participant-level dataset, and statistical code	11, 14
**Appendices**			
Informed consent materials	32	Model consent form and other related documentation given to participants and authorised surrogates	on request
Biological specimens	33	Plans for collection, laboratory evaluation, and storage of biological specimens for genetic or molecular analysis in the current trial and for future use in ancillary studies, if applicable	7-8

## Abbreviations

BSWE: Occupational self-efficacy expectations – short scale (ger.: berufliche selbstwirksamkeitserwartung)
CSSRI: Client sociodemographic and service receipt inventory
ERI: Effort reward imbalance questionnaire
EQ-5D 5 L: EuroQol’s questionnaire for health related quality of life
HADS: Hospital anxiety and depression scale
HRV: Heart rate variability
ICEA: Incremental cost-effectiveness analyses
ICER: Incremental costs effectiveness ratios
IS: Cognitive and emotional irritation scale
KFZA: Short questionnaire for work analysis (ger.: kurzfragebogen zur arbeitsanalyse)
LMX-7: Leader-member exchange 7
MANOVA: Multivariate analysis of variance
OPD-SF: Operationalized psychodynamic diagnosis structure questionnaire
PSC-4: Psychosocial safety climate questionnaire 4
PSRS: Perceived stress-reactivity-scale
SME: Small and medium-sized enterprises
WHO-5: World health Organization well-being index
QALY: Quality adjusted life years

## References

[1] Lovelace KJ, Manz CC, Alves JC (2007). Work stress and leadership development: the role of self-leadership, shared leadership, physical fitness and flow in managing demands and increasing job control. Hum Resour Manag Rev.

[2] Mulfinger N, Sander A, Stuber F, Brinster R, Junne F, Limprecht R, Jarczok MN, Seifried-Dubon T, Rieger MA, Zipfel S, Peters M, Stiawa M, Maatouk I, Helass M, Nikendei C, Rothermund E, Hander N, Ziegenhain U, Gulde M, Genrich M, Worringer B, Kullenberg J, Blum K, Suss S, Gesang E, Ruhle S, Muller A, Schweitzer-Rothers J, Angerer P, Gundel H (2019). Cluster-randomised trial evaluating a complex intervention to improve mental health and well-being of employees working in hospital - a protocol for the SEEGEN trial. BMC Public Health.

[3] Armon G, Melamed S, Shirom A, Shapira I (2010). Elevated burnout predicts the onset of musculoskeletal pain among apparently healthy employees. J Occup Health Psychol.

[4] Banerjee S, Mehta P (2016). Determining the antecedents of job stress and their impact on job performance: A study among faculty members. IUP J Organ Behav.

[5] Beehr TA, Walsh JT, Taber TD (1976). Relationships of stress to individually and organizationally valued states: higher order needs as a moderator. J Appl Psychol.

[6] Mathieu C, Fabi B, Lacoursière R, Raymond L (2016). The role of supervisory behavior, job satisfaction and organizational commitment on employee turnover. J Manag Organ.

[7] Schmidt B, Schneider M, Seeger P, van Vianen A, Loerbroks A, Herr RM (2019). A comparison of job stress models: associations with employee well-being, absenteeism, Presenteeism, and resulting costs. J Occup Environ Med.

[8] Hassard J, Teoh KR, Visockaite G, Dewe P, Cox T (2018). The cost of work-related stress to society: A systematic review. J Occup Health Psychol.

[9] Kivimäki M, Kawachi I (2015). Work stress as a risk factor for cardiovascular disease. Curr Cardiol Rep.

[10] Dragano N, Siegrist J, Nyberg ST, Lunau T, Fransson EI, Alfredsson L, Bjorner JB, Borritz M, Burr H, Erbel R (2017). Effort–reward imbalance at work and incident coronary heart disease: a multicohort study of 90,164 individuals. Epidemiology..

[11] Herr RM, Bosch JA, Loerbroks A, van Vianen AEM, Jarczok MN, Fischer JE, Schmidt B (2015). Three job stress models and their relationship with musculoskeletal pain in blue- and white-collar workers. J Psychosom Res.

[12] Madsen IE, Nyberg ST, Hanson LM, Ferrie JE, Ahola K, Alfredsson L, Batty GD, Bjorner JB, Borritz M, Burr H (2017). Job strain as a risk factor for clinical depression: systematic review and meta-analysis with additional individual participant data. Psychol Med.

[13] Aronsson G, Theorell T, Grape T, Hammarström A, Hogstedt C, Marteinsdottir I, Skoog I, Träskman-Bendz L. Hall C. A systematic review including meta-analysis of work environment and burnout symptoms. BMC Public Health. 2017;17(1) 10.1186/s12889-017-4153-7. 10.1186/s12889-017-4153-7 PMC5356239 28302088

[14] Kessler RC, Greenberg PE (2002). The economic burden of anxiety and stress disorders. Neuropsychopharmacology..

[15] Jarczok MN, Jarczok M, Thayer JF, Theorell T (2020). Work stress and autonomic nervous system activity. Handbook of Socioeconomic Determinants of Occupational Health - From Macro-level to Micro-level Evidence.

[16] Mauss D, Li J, Schmidt B, Angerer P, Jarczok MN. Work-related stress and the Allostatic load index - A systematic review. Das Gesundheitswesen. 2015; 10.1055/s-0035-1555951. 10.1055/s-0035-1555951 26402382

[17] O’Connor DB, Thayer JF, Vedhara K. Stress and health: A review of psychobiological processes. Annu Rev Psychol. 2021;72(1) 10.1146/annurev-psych-062520-122331. 10.1146/annurev-psych-062520-122331 32886587

[18] Li J, Jarczok MN, Loerbroks A, Schöllgen I, Siegrist J, Bosch JA, Wilson MG, Mauss D, Fischer JE (2013). Work stress is associated with diabetes and prediabetes: cross-sectional results from the MIPH industrial cohort studies. Int J Behav Med.

[19] Jandackova VK, Koenig J, Jarczok MN, Fischer JE, Thayer JF (2017). Potential biological pathways linking type-D personality and poor health: A cross-sectional investigation. PLoS One.

[20] Schmidt B, Herr RM, Jarczok MN, Baumert J, Lukaschek K, Emeny RT, Ladwig K-H, Investigators KORA. Lack of supportive leadership behavior predicts suboptimal self-rated health independent of job strain after 10 years of follow-up: findings from the population-based MONICA/KORA study. Int Arch Occup Environ Health. 2018; 10.1007/s00420-018-1312-9. 10.1007/s00420-018-1312-9 29687327

[21] Franke F, Ducki A, Felfe J, Felfe J (2015). Gesundheitsförderliche Führung.

[22] Arnold KA (2017). Transformational leadership and employee psychological well-being: A review and directions for future research. J Occup Health Psychol.

[23] Tafvelin S, Armelius K, Westerberg K (2011). Toward understanding the direct and indirect effects of transformational leadership on well-being: A longitudinal study. J Leadersh Org Stud.

[24] Winkler E, Busch C, Clasen J, Vowinkel J (2014). Changes in leadership behaviors predict changes in job satisfaction and well-being in low-skilled workers: A longitudinal investigation. J Leadersh Org Stud.

[25] Diebig M, Poethke U, Rowold J (2017). Leader strain and follower burnout: exploring the role of transformational leadership behaviour. Ger J Hum Resour Manage.

[26] Schyns B, Schilling J (2013). How bad are the effects of bad leaders? A meta-analysis of destructive leadership and its outcomes. Leadersh Q.

[27] Baillien E, Neyens I, De Witte H (2011). Organizational correlates of workplace bullying in small-and medium-sized enterprises. Int Small Bus J.

[28] Dawkins S, Martin A, Kilpatrick M, Scott J (2018). Reasons for engagement: SME owner-manager motivations for engaging in a workplace mental health and wellbeing intervention. J Occup Environ Med.

[29] Garavan T, Watson S, Carbery R, O’Brien F (2016). The antecedents of leadership development practices in SMEs: the influence of HRM strategy and practice. Int Small Bus J.

[30] Bernhard F, Jaskiewicz P (2011). Ownership perceptions in family businesses: psychological difficulties of the retiring owner-manager.

[31] Johnson D (1995). Stress and stress management among owner-managers of small and medium-sized enterprises.

[32] Sy T, Cote S, Saavedra R (2005). The contagious leader: impact of the leader's mood on the mood of group members, group affective tone, and group processes. J Appl Psychol.

[33] Meggeneder O (2007). Style of management and the relevance for workplace health promotion in small and medium sized enterprises. J Public Health.

[34] Joyce S, Modini M, Christensen H, Mykletun A, Bryant R, Mitchell PB, Harvey SB (2016). Workplace interventions for common mental disorders: a systematic meta-review. Psychol Med.

[35] LaMontagne AD, Martin A, Page KM, Reavley NJ, Noblet AJ, Milner AJ, Keegel T, Smith PM (2014). Workplace mental health: developing an integrated intervention approach. BMC Psychiatry.

[36] Hughes MC, Patrick DL, Hannon PA, Harris JR, Ghosh DL (2011). Understanding the decision-making process for health promotion programming at small to midsized businesses. Health Promot Pract.

[37] Richardson KM, Rothstein HR (2008). Effects of occupational stress management intervention programs: a meta-analysis. J Occup Health Psychol.

[38] Cocker F, Martin A, Scott J, Venn A, Sanderson K (2012). Psychological distress and related work attendance among small-to-medium enterprise owner/managers: literature review and research agenda. Int J Ment Health Promot.

[39] Bundesamt S (2018). Produzierendes Gewerbe und Dienstleistungen im Überblick. Statistisches Jahrbuch 2018.

[40] Hansen E, Landstad BJ, Gundersen KT, Vinberg S (2016). Leader-based workplace health interventions—a before–after study in Norwegian and Swedish small-scale enterprises. Int J Disabil Manag.

[41] Martin A, Kilpatrick M, Scott J, Cocker F, Dawkins S, Brough P, Sanderson K (2020). Protecting the mental health of small-to-medium enterprise owners: a randomized control trial evaluating a self-administered versus telephone supported intervention. J Occup Environ Med.

[42] Gray DE, Ekinci Y, Goregaokar H (2011). Coaching SME managers: business development or personal therapy? A mixed methods study. Int J Hum Resour Manag.

[43] Rind E, Emerich S, Preiser C, Tsarouha E, Rieger MA, ImproveJob Consortium (2020). Exploring drivers of work-related stress in general practice teams as an example for small and medium-sized enterprises: protocol for an integrated ethnographic approach of social research methods. JMIR Res Protoc.

[44] Arends I, Bültmann U, van Rhenen W, Groen H, van der Klink JJ (2013). Economic evaluation of a problem solving intervention to prevent recurrent sickness absence in workers with common mental disorders. PLoS One.

[45] Hamberg-van Reenen HH, Proper KI, van den Berg M (2012). Worksite mental health interventions: a systematic review of economic evaluations. Occup Environ Med.

[46] Phillips R, Schneider J, Molosankwe I, Leese M, Foroushani PS, Grime P (2014). Randomized controlled trial of computerized cognitive behavioural therapy for depressive symptoms: effectiveness and costs of a workplace intervention. Psychol Med.

[47] van de Poll MK, Bergström G, Jensen I, Nybergh L, Kwak L, Lornudd C, Lohela-Karlsson M (2020). Cost-effectiveness of a problem-solving intervention aimed to prevent sickness absence among employees with common mental disorders or occupational stress. Int J Environ Res Public Health.

[48] Limm H, Gündel H, Heinmüller M, Marten-Mittag B, Nater UM, Siegrist J, Angerer P (2011). Stress management interventions in the workplace improve stress reactivity: a randomised controlled trial. Occup Environ Med.

[49] Li J, Riedel N, Barrech A, Herr RM, Aust B, Mörtl K, Siegrist J, Gündel H, Angerer P (2017). Long-term effectiveness of a stress management intervention at work: A 9-year follow-up study based on a randomized wait-list controlled trial in male managers. Biomed Res Int.

[50] Commssion of the european communities (2003). Commission Recommendation of 6 May 2003 concerning the definition of micro, small and medium-sized enterprises.

[51] IfM Bonn (2016). KMU-Definition des IfM Bonn: Institut für Mittelstandsforschung.

[52] Schlotz W, Yim IS, Zoccola PM, Jansen L, Schulz P (2011). The perceived stress reactivity scale: measurement invariance, stability, and validity in three countries. Psychol Assess.

[53] Rödel A, Siegrist J, Hessel A, Brähler E (2004). Fragebogen zur Messung beruflicher Gratifikationskrisen. Z Differentielle Diagnostische Psychol.

[54] Caliskan G (2015). An examination of coach and player relationships according to the adapted LMX 7 scale: A validity and reliability study. Meas Phys Educ Exerc Sci.

[55] Dollard MF, Dollard MF, Dormann C, Idris MA (2019). The PSC-4; A short PSC tool. Psychosocial safety climate.

[56] Ehrenthal J, Dinger U, Horsch L, Komo-Lang M, Klinkerfuß M, Grande T, Schauenburg H (2012). Der OPD-Strukturfragebogen (OPD-SF): Erste Ergebnisse zu Reliabilität und Validität. Psychother Psychosom Med Psychol.

[57] Roick C, Kilian R, Matschinger H, Bernert S, Mory C, Angermeyer MC (2001). Die deutsche version des client Sociodemographic service receipt inventory (CSSRI-EU). (German adaptation of the client Sociodemographic and service receipt inventory). Psychiatr Prax.

[58] Herdman M, Gudex C, Lloyd A, Janssen MF, Kind P, Parkin D, Badia X (2011). Development and preliminary testing of the new five-level version of EQ-5D (EQ-5D-5L). Qual Life Res.

[59] Ludwig K, Graf von der Schulenburg J-M, Greiner W (2018). German value set for the EQ-5D-5L. Pharmacoeconomics.

[60] Wennig R (2000). Potential problems with the interpretation of hair analysis results. Forensic Sci Int.

[61] Stalder T, Steudte S, Alexander N, Miller R, Gao W, Dettenborn L, Kirschbaum C (2012). Cortisol in hair, body mass index and stress-related measures. Biol Psychol.

[62] Nater UM, Rohleder N (2009). Salivary alpha-amylase as a non-invasive biomarker for the sympathetic nervous system: current state of research. Psychoneuroendocrinology..

[63] Strahler J, Skoluda N, Kappert MB, Nater UM (2017). Simultaneous measurement of salivary cortisol and alpha-amylase: application and recommendations. Neurosci Biobehav Rev.

[64] Skoluda N, Dhrami I, Nater UM (2020). Factors contributing to stability and instability in alpha-amylase activity in diluted saliva samples over time. Psychoneuroendocrinology..

[65] Jarczok MN, Aguilar-Raab C, Koenig J, Kaess M, Borniger JC, Nelson RJ, Fischer JE (2018). The heart’ s rhythm ‘n’blues: sex differences in circadian variation patterns of vagal activity vary by depressive symptoms in predominantly healthy employees. Chronobiol Int.

[66] Kim H-G, Cheon E-J, Bai D-S, Lee YH, Koo B-H (2018). Stress and heart rate variability: a meta-analysis and review of the literature. Psychiatry Investig.

[67] Aust F, Diedenhofen B, Ullrich S, Musch J (2013). Seriousness checks are useful to improve data validity in online research. Behav Res Methods.

[68] Prümper J, Hartmannsgruber K, Frese M (1995). KFZA. Kurz-Fragebogen zur Arbeitsanalyse. Z Arbeits-Und Organisationspsychologie.

[69] Appel P, Schuler M, Vogel H, Oezelsel A, Faller H (2017). Short questionnaire for workplace analysis (KFZA): factorial validation in physicians and nurses working in hospital settings. J Occup Med Toxicol.

[70] Mohr G, Rigotti T, Müller A (2005). Irritation-ein instrument zur Erfassung psychischer Beanspruchung im arbeitskontext. Skalen-und itemparameter aus 15 Studien. Z Arbeits-und Organisationspsychologie.

[71] Topp CW, Østergaard SD, Søndergaard S, Bech P (2015). The WHO-5 well-being index: a systematic review of the literature. Psychother Psychosom.

[72] Read BL (2018). Serial interviews: when and why to talk to someone more than once. Int J Qual Methods.

[73] Mayring P, Mey G, Mruck K (2010). Qualitative Inhaltsanalyse. Handbuch Qualitative Forschung in der Psychologie.

[74] Petermann F. Hospital anxiety and depression scale, deutsche version (HADS-D). Z Psychiatr Psychol Psychother. 2015;59(3).

[75] Sischka PE, Costa AP, Steffgen G, Schmidt AF (2020). The WHO-5 well-being index–validation based on item response theory and the analysis of measurement invariance across 35 countries. J Affect Disord Rep.

[76] Glick HA, Doshi JA, Sonnad SS, Polsky D (2014). Economic evaluation in clinical trials.

[77] Salize H-J, Kilian R (2010). Gesundheitsökonomie in der Psychiatrie. Konzepte, Methoden, Analysen.

[78] Willan AR, Briggs AH (2006). Statistical analysis of cost-effectiveness data.

[79] Skakon J, Nielsen K, Borg V, Guzman J (2010). Are leaders’ well-being, behaviours and style associated with the affective well-being of their employees? A systematic review of three decades of research. Work Stress.

[80] Martin A, Kilpatrick M, Cocker F, Sanderson K, Scott J, Brough P (2015). Recruitment and retention challenges of a mental health promotion intervention targeting small and medium enterprises. Derailed Organizational Interventions for Stress and Well-Being.

[81] Chan AW, Tetzlaff JM, Altman DG, Laupacis A, Gøtzsche PC, Krleža-Jerić K, Hróbjartsson A, Mann H, Dickersin K, Berlin JA, Doré CJ, Parulekar WR, Summerskill WS, Groves T, Schulz KF, Sox HC, Rockhold FW, Rennie D, Moher D (2013). SPIRIT 2013 statement: defining standard protocol items for clinical trials. Ann Intern Med.

